# Fees for Dental Pupilage

**Published:** 1865

**Authors:** C. M. Wright


### FEES FOR DENTAL PUPILAGE.

BY C. M. WRIGHT.

Read before the Cincinnati Dental Society.

GENTLEMEN: I will not presume or attempt to instruct or even entertain so learned a body as the Cincinnati Dental Association, and, therefore, have turned my back on scientific matters and the subject chosen for discussion this evening. In regard to many of the important questions brought before and so ably disposed of by this society, I, of course, have opinions, and am forming opinions; but in committing to paper thoughts upon such subjects as "The Reproduction of Bone," (with or without the assistance of a periosteum,) "Sensitive Dentine," "Periostitus," and others of equal importance, a natural caution makes me doubt the solidity of the ground upon which I sometimes stand, and fear the consequences of getting beyond my depth. Then, lest I should be drowned, or, at least, flounder about so in the deep waters of these deep subjects, I beg leave to suggest a matter which I cannot but think worthy of some attention by members of our profession. It is now the custom, I believe, among many dentists to charge from one to five hundred dollars for "dental instruction" to any young man whose tastes or hopes are for the practice of dentistry. Many of these gentlemen themselves would never have been in the profession, perhaps, if this bar of "a fee" had been placed in their way. It seems to me to be not only an injustice to the student and to the dental profession, but an act which might, with propriety, be termed "unprofessional." It does not seem to me to be in accordance with the spirit of so many essays and speeches now floating about in our periodicals, about Popular Education, People's Journals, Instructing Patients, &c. It places the dentist in the light of a juggler, whose arts and tricks will be taught or sold for a consideration. The dentist, like the veiled prophet of Kerassen, has arts which he practices, and which he scrupulously hides from the public eye; but, unlike the old prophet, he will disclose his ugly phiz for a hundred dollars. When a dentist proclaims that he has methods of practice unknown to the profession—when he extracts teeth by a new and original process, or plugs with a new material, and refuses to open his laboratory or operating rooms to his professional brothers, or refuses to explain the relation existing between himself and the unclean spirits of the air who assist him, he is immediately and justly pronounced a charlatan, and silently, even tenderly, dropped from his professional standing among dentists. But when a dentist, in full practice, who has need of an assistant, for malleting, and for the dirty sand and plaster, zinc and lead-work of his laboratory, agrees to permit a young man to come into his office and do all he can to assist his preceptor for a hundred or even five hundred dollars, he is, no doubt, performing a noble work for his beloved profession. A dentist should not, in my opinion, take a student, unless he is satisfied that the applicant is fitted for a dental student; and if he is capable of making a dentist of himself, he will be of use to his preceptor in a very few months. Then, a dentist should not admit a student, unless he expects to devote himself faithfully to the instruction of that student. He should not only direct his reading, but "quiz" the pupil thoroughly. He should offer as many opportunities as possible for his student to witness operations; and work done by the student should be carefully criticised.

It is not expected, however, that a man with a large practice will give up his patients or neglect his engagements for the benefit of any young man; and it is not often the case, even if five hundred dollars have been paid as the pupilage fee. Five hundred—even a thousand dollars—will not pay a good dentist for time devoted to his student, if he neglects his patients; but then if we only work for *fees*, why will we talk and write so much about our love for our adored profession—about our anxiety for its rapid advancement—about our duties to our chosen profession, aside from greenback considerations—about the necessity for sacrifices and god-like energy from each and every member of our honorable body for the sole benefit of his profession? Why are we interested in our dental colleges, our dental societies? Why do we demand a high standard of attainments from members, and write papers for our periodicals about the duties of students and the advantage of dentistry, and laugh to scorn, or point the finger of contempt at the man who regards a *fee* more than he does a good operation? Why do we do and say all this, and yet demand a property qualification of our students? Yes, sir, we say to a young man with his mind bent on dentistry, "We think you possess first-rate abilities; you have a good education; you are something of a mechanic; you seem to have a noble ambition and right feelings about dentistry; but have you got a hundred dollars for our *fee?* If you have not, you'd better turn your attention to shoemaking or school-teaching; you'll never do for a professional gentleman." Of course, a good dental instruction is worth many thousands of dollars to a student; but then do we not always pocket a fee of this kind as a clear gain, with the secret satisfaction that we have, at the same time, secured an errand-boy and an assistant in the bargain!

Gentlemen, I did not commence this with the intention of saying so much, or of arguing the case. I desire simply, if possible, to provoke an opinion, or opinions from others better calculated to do the subject justice.

